# Bad Cholesterol Uptake by CD36 in T-Cells Cripples Anti-Tumor Immune Response

**DOI:** 10.20900/immunometab20210028

**Published:** 2021-09-14

**Authors:** Mikhail G. Kolonin

**Affiliations:** The Brown Foundation Institute of Molecular Medicine, University of Texas Health Science Center, Houston, TX 77030, USA

**Keywords:** T-cells, anti-tumor immune response, cholesterol

## Abstract

Despite the advances in immunotherapies, effective against some types of cancer, progression of several types of carcinoma remains uncurable. Recent studies indicate that changes in lipid metabolism, aggravated by obesity, disable anti-tumor immune response. In the July issue of *Immunity*, Xu et al. use mouse models to demonstrate that certain types of oxidized lipids, transported by CD36, suppress the capacity of CD8^+^ T lymphocytes to secrete cytotoxic molecules. This study sheds light on how lipid modifications in the tumor microenvironment make killer T cells incapable of inhibiting tumor growth.

Anti-tumor immune response can become inadequate as a result of the immunosuppressive tumor microenvironment. While cytotoxic CD8^+^ T cells typically infiltrate tumors, they become unable to effectively execute their effector (killer) function. This reduces the efficacy of immunotherapies, such as PD1 immune checkpoint blockade. Better understanding of the mechanisms that inhibit antitumor immunity and identification of new potential therapeutic strategies are the hot pursuits in immuno-oncology.

Lipid metabolism plays important roles in both T lymphocyte differentiation and cancer cell survival. In the July issue of Immunity, Xu et al. report that uptake of oxidized lipids, mediated by CD36, in CD8^+^ T lymphocytes leads to their dysfunction and escalated cancer progression [1]. This collaborative study, led by Dr. Susan Kaech, has found that in tumors killer T cells accumulate oxidized lipids and switch to expressing immune-suppressive cell surface molecules and cytokines. This is specifically observed in T cells expressing high levels of CD36, a lipid transporter, indicating that these lipids are taken up from the outside rather than generated through lipogenesis. The scavenger receptor CD36, also known as FA translocase (FAT) is known to facilitate the transport of various long chain fatty acid and cholesterol species [2,3]. Oxidized low-density lipoproteins (LDLs), the ‘bad LDL’ also linked with atherosclerosis [4], as well as phospholipids, were found to be the main types of lipids accumulating in tumor-infiltrating lymphocytes. The importance of lipid peroxidation in generation of these immunosuppressive lipids was demonstrated by experiments in which GPX4, a glutathione peroxidase removing oxidized lipids, was ectopically expressed in T cells. The study shows that the damage in T cells is mediated by a stress response protein kinase p38 [1].

CD36 is expressed in various cell types and is known to mediate lipid transport in tumors [5]. While downregulation of CD36 has been observed in aggressive breast cancers [6], there is accumulating evidence that cancer progression can be promoted by lipids hijacked from circulation and acquired by cancer cells [7,8]. Recent evidence indicates a key role of fatty acids released by lipolytic adipocytes in fat tissue surrounding carcinomas [9–11]. Our recent report indicates an importance of CD36 in the release of long-chain fatty acids (LCFAs) from adipose tissue and their utilization for beta-oxidation in carcinoma cells [12]. Here, Xu et al. demonstrate a reduced growth of subcutaneously grafted MC38 adenocarcinoma and B16 melanoma tumors in mice lacking CD36 systemically. Suppression of tumor growth, linked with increased pro-inflammatory cytokine expression, was also observed upon mouse treatment with a CD36-blocking antibody. Because the effects of systemic CD36 inactivation could be due to lipid transport blockade in various cell types, the authors demonstrated the specific importance of CD36 expressed by killer T cells through adoptive transfer. Transplantation of CD36-null CD8^+^ T cells resulted in increased T cell response and reduced tumor growth, compared to CD36^+^ T cells.

Consistent with results of Xu et al., a recent preceding study, based on different mouse cancer models, reported that CD36-mediated cell death dampens intratumoral CD8^+^ T cell effector function and impairs their antitumor activity [13]. Specifically, programmed cell death dependent on iron and characterized by the accumulation of lipid peroxides (ferroptosis) was found to occur in CD36-expressing tumor-infiltrating CD8^+^ T cells. Both studies revealed that ferroptosis in CD36^+^ T cells was linked with reduced levels of cytotoxic cytokines. As in the study by Xu et al., CD36 upregulation was observed selectively in tumor-infiltrating lymphocytes. While the suppressive effect of LDL and phospholipid oxidation on killer T cells is clear, experiments with labeled LCFAs did not reveal significant differences [1]. However, there are conceptual and technical caveats with using specific LCFA species for such experiment. A trend for lower neutral lipid content in CD36-null cells in this study suggests that LCFA transport may be important and its contribution to CD36-mediated metabolic pathways remains to be determined.

Other CD36-mediated pathways effecting their function and cancer progression also cannot be excluded. Lipid metabolism controls differentiation and functions of various types of T lymphocytes, and ultimately the maintenance of immune tolerance [14]. While the study by Xu et al. demonstrates the role of CD36-mediated lipids on the function and survival of killer T-cells, the possible effects on their generation and tumor recruitment remain to be investigated. Considering the effects of CD36-mediated lipid transport on other cells may be as important. In macrophages, oxidated LDL interaction with CD36 mediates the activation of NF-κB and promotes inflammation. However, binding of the apoptotic cells to CD36 on macrophages promotes anti-inflammatory cytokine response [4]. It is possible that in T cells CD36-mediated pathways may also have context-dependent effects. Indeed, an opposite effect of CD36 expression on immunosuppressive T_reg_ cells has been reported [15]. In that study, Wang et al. reported that CD36-mediated lipid metabolism activation in T_reg_ cells promotes their survival, accumulation in the tumor, and disabling of the antitumor CD8^+^ T cell response. In addition, CD36 is expressed by dendritic cells and mediates antigen presentation, which is important to consider in the context of cancer immunology [16].

The paucity of CD36 molecular interactions within each cell type further complicates the interpretation of phenotypes resulting from its inactivation. Because CD36 also plays a role in angiogenesis and inflammation [17], alterations in these processes and their potential effect on cancer progression cannot be excluded. For example, CD36 binds thrombospondins, which mediate cell adhesion and migration by regulating cell-to-cell and cell-to-matrix interactions. Specifically, CD36 modulates the thrombospondin antiangiogenic activity, which has obvious implications on tumor growth [3]. In addition, in myocytes and other cell types, CD36 activates AMPK signaling. The importance of CD36 in regulating this pivotal cell survival pathway is yet to be assessed in immune cells. Finally, CD36 has been reported to form a complex with annexin-A2 and prohibitin-1 [18], both of which have reported roles in cancer [19,20]. Because a recent study demonstrates a particular importance of the CD36 fatty acid transporter function in the context of high fat diet feeding [12], the potential importance of diet for the CD36-mediated effects on immune cells remains to be determined.

These considerations raise practical questions. While immune checkpoint blockade therapy has revolutionized treatment of some cancers, it has limited efficacy in many types of carcinoma. Although CD36 inactivation in killer T cells could bolster the anti-tumor effect, it is not clear how its systemic targeting in other cell types might affect disease progression long-term. Importantly, activation of lipid oxidation in cancer cells, leading to cytotoxicity, has been considered as an approach to cancer treatment. Therefore, establishing selective approaches to activating lipid oxidation in cancer cells, while suppressing it in killer T cells, would be needed. Despite these challenges, the study by Xu et al. is an important new step toward the development of more effective immunotherapies for cancer.
